# Comparative studies on the room-temperature ferrielectric and ferrimagnetic Ni_3_TeO_6_-type A_2_FeMoO_6_ compounds (A = Sc, Lu)

**DOI:** 10.1038/srep20133

**Published:** 2016-02-01

**Authors:** Guang Song, Weiyi Zhang

**Affiliations:** 1 National Laboratory of Solid State Microstructures and Department of Physics, Nanjing University, Nanjing 210093, China; 2 Collaborative Innovation Center of Advanced Microstructures, Nanjing University, Nanjing 210093, China

## Abstract

First-principles calculations have been carried out to study the structural, electric, and magnetic properties of Ni_3_TeO_6_-type A_2_FeMoO_6_ compounds (A = Sc, Lu). Their electric and magnetic properties behave like room-temperature ferrielectric and ferrimagnetic insulators where polarization comes from the un-cancelled antiparallel dipoles of (A(1), Fe^3+^) and (A(2), Mo^3+^) ion groups, and magnetization from un-cancelled antiparallel moments of Fe^3+^ 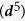 and Mo^3+^ 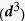 ions. The net polarization increases with A’s ionic radius and is 7.1 and 8.7 *μ*Ccm^−2^ for Sc_2_FeMoO_6_ and Lu_2_FeMoO_6_, respectively. The net magnetic moment is 2 **μ**_***B***_ per formula unit. The magnetic transition temperature is estimated well above room-temperature due to the strong antiferromagnetic superexchange coupling among Fe^3+^ and Mo^3+^ spins. The estimated paraelectric to ferrielectric transition temperature is also well above room-temperature. Moreover, strong magnetoelectric coupling is also anticipated because the magnetic ions are involved both in polarization and magnetization. The fully relaxed Ni_3_TeO_6_-type A_2_FeMoO_6_ structures are free from soft-phonon modes and correspond to stable structures. As a result, Ni_3_TeO_6_-type A_2_FeMoO_6_ compounds are possible candidates for room-temperature multiferroics with large magnetization and polarization.

Single phase polar materials with ferromagnetic (ferrimagnetic) properties have drawn much attention^1^,^2^,^3^ recently due to their applications in developing spintronic devices for nonvolatile memories and in achieving electric-field control of magnetization in realistic information storage^4^,^5^,^6^,^7^. Therefore, searching for multiferroic materials becomes an important research direction in material physics. Up to now, various mechanisms have been proposed to explain the electric polarization in magnetic compounds. Among others, the off-center displacement of lone-pairs 6*s* electrons^8^,^9^, the chiral spin-density-wave driven polarization^10^,^11^,^12^, the charge ordering^13^,^14^,^15^, and the strain-induced polarization are mechanisms being discussed most^16^,^17^,^18^,^19^. Although great progresses have been made in developing single phase multiferroic materials, many important issues remain unsolved. For example, compounds with both large magnetization and polarization are still rare; the ferro(ferri)magnetic transition temperatures are usually below room temperature and restricted their applications; even if the requirements of large magnetization and polarization are fulfilled, enhancing magnetoelectric coupling is still a big challenge.

In order to meet these crucial requirements, searching for the multiferroics which have magnetic ions contributing simultaneously to electric polarization can be a good choice. Thus in this report, we have analyzed the structural, electric, and magnetic properties of two corundum-derived oxides A_2_FeMoO_6_ (A = Sc, Lu). All of them are found to be multiferroic materials and have the same polar structure as Ni_3_TeO_6_^3^, ZnTiO_3_^20^, and FeTiO_3_^21^. The general crystal structure displayed in Fig. 1(a) is described by a chemical formula 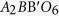 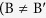 for Ni_3_TeO_6_ type; 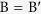 for LiNbO_3_ or FeTiO_3_ type). The structural advantage is its ability to incorporate different magnetic transition-metal ions on all cation sites for realizing magnetoelectric coupling. The common feature of the structures is the small A-site cation in six oxygen coordination, such as Sc^3+^ (0.69 Å) and Lu^3+^ (0.745 Å), in comparison with the large alkaline earth cation in twelve oxygen coordination, such as Ca^2+^ (1.34 Å), Sr^2+^ (1.44 Å), and Ba^2+^ (1.61 Å). The distortion of the structure can be estimated by a similar tolerance factor defined for a perovskite compound, 
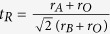
, where 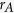, 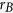, and 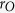 are the ionic radii of A-site ion, B-site ion (in 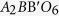, 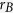 is an averaged radius of B- and B′-site ions)^22^, and O ions, respectively. As found in most systems, for 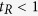, the cubic perovskite changes its symmetry by the BO_6_ octahedral rotation or tilting or Jahn-Teller distortion. Good examples are the Ni_3_TeO_6_ (Ni_2_NiTeO_6_) compound with nonhysteretic colossal magnetoelectricity^3^, ScFeO_3_ (Fe takes both the B- and B′-sites)^23^,^24^ and Mn_2_FeMO_6_ (M = Nb, Ta, Mo, and W)^25^,^26^,^27^ compounds with polar structure and antiferromagnetic or ferrimagnetic structure. Due to the strong antiferromagnetic superexchange coupling between nearest neighbors Fe^3+^ 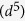 in ScFeO_3_, the Néel temperature is well above room-temperature (545 K). To improve the net magnetization of ScFeO_3_, constructing ferrimagnetic structure by replacing one of the B-site Fe^3+^ by a *d*^*n*^ (n < 5) ion is a possible way, which was done in the synthesized Bi_2_FeCrO_6_ (Cr^3+^: *d*^3^ compound^28^,^29^).

## Results

To accomplish this goal, we have carried out comprehensive first-principles study on Ni_3_TeO_6_–type A_2_FeMoO_6_ compounds (A = Sc, Lu) where one of the B-site Fe^3+^ is replaced by Mo^3+^ 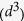. The structural, electric, and magnetic properties of Ni_3_TeO_6_–type A_2_FeMoO_6_ have been systematically analyzed as a function of A-site cation radius. We found that the ferrimagnetic state is indeed the ground-state with net magnetic moment of 2 μ_*B*_/f.u. forming between the antiparallel Fe^3+^ (5 μ_*B*_) and Mo^3+^ (3 μ_*B*_) ions. The polarization increases with A’s ionic radius and is 7.1 and 8.7 *μ*Ccm^−2^ for Sc_2_FeMoO_6_ and Lu_2_FeMoO_6_, respectively. Moreover, strong magnetoelectric coupling is achieved since the electric polarization comes partly from the same magnetic ions. The robust antiferromagnetic coupling is sustained, ensuring a Néel temperature well above room temperature. The structural analyses suggest that Ni_3_TeO_6_–type A_2_FeMoO_6_ compounds are free from soft-phonon modes and correspond to stable structures.

## Discussion

**Figure 1:**
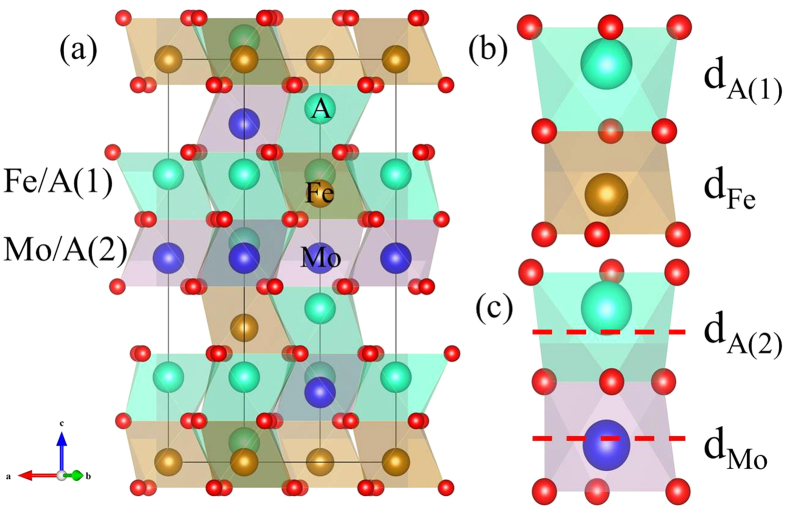
The crystal structure of Ni_3_TeO_6_-type A_2_FeMoO_6_ with *R3* space group. (**a**) Structure viewed along 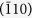 direction. (**b**) face-sharing A(1)O_6_/FeO_6_ octahedral pair. (**c**) face-sharing A(2)O_6_/MoO_6_ octahedral pairs. The dashed lines refer to the neutral plane of oxygen octahedron along c-axis and d denotes the displacement for various transition metal ions. The spheres for different ions are also indicated.

Let us start with the Ni_3_TeO_6_-type structures of A_2_FeMoO_6_ compounds as shown in Fig. 1(a). The structures are obtained after the full relaxation of lattice parameters and atomic positions with effective on-site Coulomb repulsion 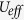 = 4.0, 1.0, and 5.0 eV for Fe-3d, Mo-4d and Lu-5f electrons, respectively. The structures can be constructed in two steps: (1) (A(1)O_6_, FeO_6_) (Fig. 1(b)) and (A(2)O_6_, MoO_6_) (Fig. 1(c)) octahedral pairs form face-sharing structures along *c*-axis; (2) the two face-sharing structures then form zigzag chains by edge-sharing A(1)O_6_/FeO_6_ and A(2)O_6_/MoO_6_ octahedral pairs in the *ab*-plane. Due to the strong electrostatic repulsion among the neighboring cations in the centers of the face-shared octahedral pairs, large antiferro-polar displacements take place along *c*-axis for (A(1), Fe), and (A(2), Mo) ion pairs (see Fig. 1(b,c)). Thus, antiparallel electric moments are formed for each face-sharing A(1)O_6_/FeO_6_ and A(2)O_6_/MoO_6_ octahedral pairs, and ferrielectric polarization is generated along *c*-axis. The fully optimized structural parameters and atomic positions of ferrimagnetic Ni_3_TeO_6_-type A_2_FeMoO_6_ are listed in Table 1 together with those of antiferromagnetic ScFeO_3_ as a reference. In Table 1, the lattice parameters, atomic positions, and bond angles are highly accurate. A relative error less than 1% is achieved between our calculated data and the available experimental data^23^,^24^. The spontaneous polarization was computed using the Berry phase method^30^. The total polarization of ScFeO_3_ is 2.0 and 1.6 *μ*Ccm^−2^ for theoretically optimized and the experimentally measured structures, respectively. These results are close to the value 1.4 *μ*Ccm^−2^ observed experimentally^23^. The computed polarization is 7.1 and 8.7 *μ*Ccm^−2^ for ferrimagnetic Sc_2_FeMoO_6_ and Lu_2_FeMoO_6_, respectively. The polarization increases with A’s ionic radius. Larger radius, probably, strengthens the repulsive force between neighboring ions in the centers of face-sharing A(1)O_6_/FeO_6_ and A(2)O_6_/MoO_6_ octahedral pairs. Our study shows that the ferrimagnetic structures not only greatly improved the magnetization property, but also significantly enhanced the polarization of A_2_FeMoO_6_ regarding the reference compound ScFeO_3_. The incompatibility between ferroelectricity and ferromagnetism gets nicely reconciled in the ferrielectric and ferrimagnetic A_2_FeMoO_6_^31^. In addition, strong magnetoelectric coupling between the polarization and magnetization is also intrinsically embedded in the structures.

**Figure 2:**
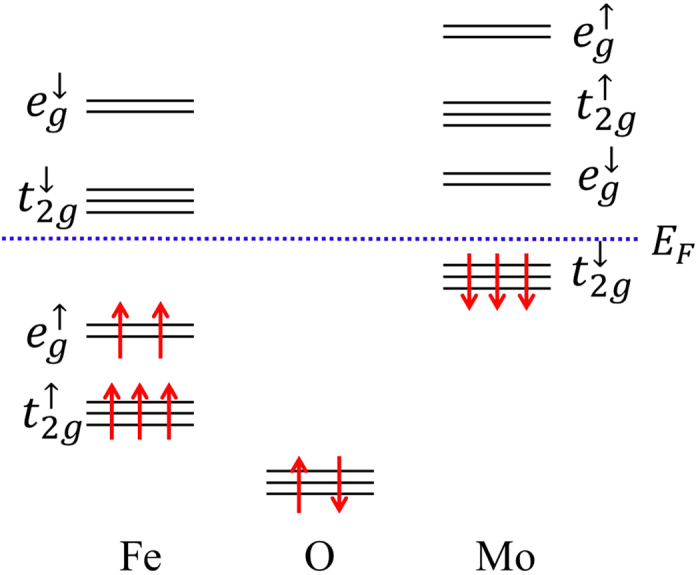
Schematic diagram for the atomic energy levels of Fe-*d*, Mo-*d* and O-*p* orbitals. The small arrows denote the spin states while the red large arrows refer to the occupied electron spins. The horizontal dashed line refers to Fermi energy.

Having investigated the structural and electric properties of Ni_3_TeO_6_-type A_2_FeMoO_6_ compounds, we are now in position to discuss their electronic and magnetic properties. For Ni_3_TeO_6_-type A_2_FeMoO_6_, the orbital configurations of 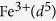 and 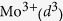 are similar to those of La_2_FeCrO_6_ according to previous study^32^. The schematic diagram for the relevant atomic energy levels is illustrated in Fig. 2. The spin-up and spin-down *d*-orbitals are separated by spin exchange energy Δ_*S*_, *d*(*e*_*g*_) and *d*(*t*_2*g*_) orbitals are separated by a crystal-field-splitting energy 10*Dq*. The nature of the superexchange coupling between 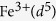 and 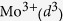 ions is quite complicated because of the orbital degeneracy and two possible hybridization schemes. pdσ hopping favors ferromagnetic superexchange coupling while pdπ hopping favors antiferromagnetic superexchange coupling. The subtle competition between the two determines the magnetic ordering of ground state. Our first-principles calculations show that the ferrimagnetically ordered state is consistently lower in energy than that of the ferromagnetically ordered state in Ni_3_TeO_6_-type A_2_FeMoO_6_. Thus the polar state with ferrimagnetic ordering can be the favored ground state.

**Figure 3:**
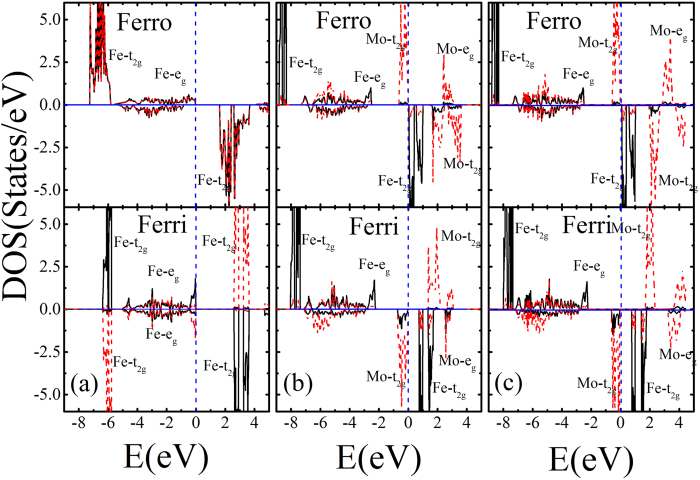
The spin and atom (Fe, Mo) projected densities of states of ScFeO_3_ and A_2_FeMoO_6_ with 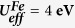, 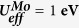, and 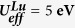. The DOSs for ferromagnetic and ferrimagnetic states are presented as an upper-half and lower-half of each sub-figure. The spin-up and spin-down DOSs are plotted upwards and downwards respectively. The solid and dashed lines refer to the two different sites of transition metal ions (Fe, Fe for ScFeO_3_; Fe, Mo for Sc_2_FeMoO_6_ and Lu_2_FeMoO_6_). The orbital characters are indicated in the spectra. (**a**) ScFeO_3_. (**b**) Sc_2_FeMoO_6_. (**c**) Lu_2_FeMoO_6_. The dashed vertical line is the Fermi energy which is set to 0.

To have an overall picture of the electronic and magnetic properties of A_2_FeMoO_6_, the spin-resolved partial densities of states (DOS) are plotted in Fig. 3 for both ferromagnetically and ferrimagnetically ordered structures. To distinguish between the two types of transition-metal ions associated with A(1)O_6_/FeO_6_ and A(2)O_6_/MoO_6_ octahedral pairs, the DOSs of Fe and Mo are represented by solid (black) and dashed (red) lines, respectively. As shown in Fig. 3, the positions of extended 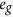 and localized 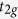 orbitals are in accord with the atomic level scheme in Fig. 2. In agreement with previous studies^23^,^24^, the ScFeO_3_ demonstrates large band gap for both the ferromagnetic 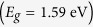 and antiferromagnetic 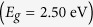 states. For A_2_FeMoO_6_ (A = Sc, Lu), the ferromagnetic state shows a vanishingly small band gap while sizeable band gap is present for the ferrimagnetic state. The band gap of ferrimagnetic state is 0.71 and 0.73 eV for Sc_2_FeMoO_6_ and Lu_2_FeMoO_6_, respectively. The overall electronic spectra are quite similar for the two different compounds regarding the partial densities of states for Fe and Mo *d*-orbitals. The 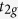 orbitals of Fe and Mo are further reduced to 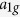 and 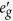 manifolds because of the trigonal crystal-field-splitting energy. Due to the strong hybridization with O 2*p* states, the *e*_*g*_ bands are rather extended. The spin exchange energy is about 2.90 eV for Fe and 1.32 eV for Mo. The total magnetic moment per formula unit is 2 μ_*B*_ in the ferrimagnetically ordered state. For ferromagnetically ordered state, the total magnetic moment per formula unit is 8 μ_*B*_. The projected magnetic moments on Fe and Mo are 4.06, 2.15 μ_*B*_ and 4.06, 2.20 μ_*B*_ for Sc_2_FeMoO_6_ and Lu_2_FeMoO_6_, respectively. These values are consistent with the high-spin configuration of Fe^3+^ and Mo^3+^. The projected magnetic moment on Fe in ScFeO_3_ is 4.15 μ_*B*_, slightly larger than the value 3.71 μ_*B*_ measured experimentally.

The electronic structural patterns can be understood from the level scheme of Fig. 2 together with hybridization processes. In particular, the valence and conduction bands near the Fermi energy is mainly resulted from the 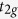 orbitals of Fe and Mo. For the ferromagnetically ordered state, the up-spin 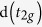 orbitals form the Fe and Mo dominated valence bands while the down-spin 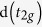 orbitals form the Fe and Mo dominated conduction bands. The hybridization with oxygen orbitals pushes the Mo dominated 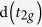 valence band edge upwards and pulls the Fe dominated 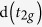 conduction band edge downwards. This makes the ferromagnetic band-gap extremely small. For the ferrimagnetically ordered state, the band structure in the vicinity of the Fermi energy is mainly determined by down-spin 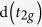 orbitals of Fe and Mo across the Fermi energy. The difference in energy level essentially determines the band-gap between Fe dominated conduction band and Mo dominated valence band. This also explains why the overall features of DOSs for A_2_FeMoO_6_ (A = Sc, Lu) look rather similar. In addition, above discussion suggests that ferrimagnetically ordered state mainly involves hybridizing down-spin 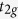 orbitals of Fe and Mo across the Fermi energy. The resulting band splitting, thus, can significantly lower the binding energy. This is also the basic mechanism dictating the ferrimagnetically ordered ground state. The similar scenario also takes place in the double perovskite La_2_FeCrO_6_ as proved by the GGA electronic structure calculation^32^.

**Figure 4:**
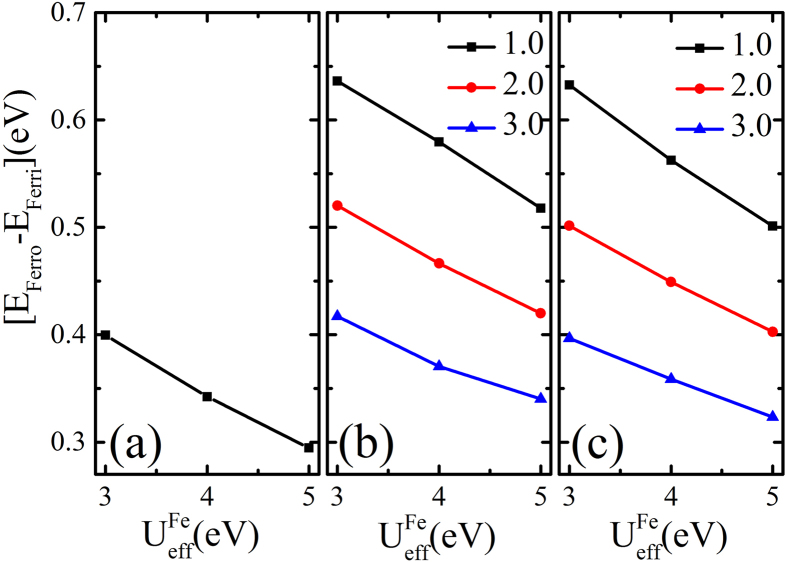
The energy difference ΔE per unit cell between ferromagnetic and ferrimagnetic states as functions of 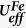. The lines denoted by solid squares, circles, and triangles refer to 
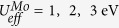
. 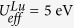. The unit cell contains two formula units for ScFeO_3_ and one formula unit for A_2_FeMoO_6_. (**a**) ScFeO_3_. (**b**) Sc_2_FeMoO_6_. (**c**) Lu_2_FeMoO_6_.

It is known that the choice of the Coulomb interaction 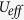 has a notable impact on the electronic structure, and thus affects the relative stability of different magnetically ordered states. To investigate such effect, we have also performed GGA + *U* simulations for other 
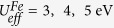
 and 
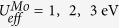
 while keeping 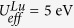. The choice of parameter values are based on the fact that the Coulomb interaction is typically weaker for spatially more extended 4*d* electrons than for more localized 3*d* electrons. The computed energy difference 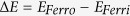 between the ferromagnetically and ferrimagnetically ordered states are shown in Fig. 4 as functions of 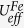 and 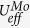. It has been found that ΔE is a monotonic deceasing function with increasing 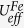 or 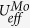, which varies from 0.65 to 0.3 eV, but the ferrimagnetically ordered state is consistently lower than that of the ferromagnetically ordered state. The monotonic decreasing behavior of energy difference originates from the superexchange interaction, 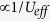, for ferrimagnetic state since the ferromagnetic state is less sensitive to 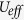. The energy difference decreases slightly as A’s ionic radius increases, because large A’s ionic radius reduces the effective hopping integral between Fe and Mo ions and so is that of the antiferromagnetic superexchange coupling. However, large A’s ionic radius expands the oxygen octahedra and favors the polar distortion. To estimate the magnetic transition temperature for A_2_FeMoO_6_ and ScFeO_3_, we adopt the single parameter Heisenberg spin model 
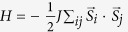
 by assuming the same exchange parameter for all the nearest-neighbor couplings. Using S = 5/2 for Fe^3+^ and S = 3/2 for Mo^3+^, one can determine the exchange coupling *J* by matching the energy differences obtained from the Heisenberg model and first-principles calculations. Then magnetic transition temperature 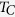 is related to the energy difference ΔE by 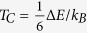. For 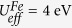, 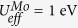, and 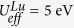 which best reproduced the experimentally observed lattice parameters, *T*_*C*_ of ScFeO_3_ is 661 K. The mean-field estimated 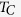
is higher than the measured value 545K^26^ since the spin fluctuation effect is not accounted for. Similar estimates yield 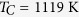 for Sc_2_FeMoO_6_ and 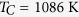 for Lu_2_FeMoO_6_, all above room-temperature. More practical estimate of 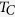 can be made by scaling the energy difference with respect to that of ScFeO_3_, which gives 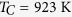 for Sc_2_FeMoO_6_ and 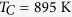 for Lu_2_FeMoO_6_. This is consistent with Lu’s results on AlScFeMoO_6_ (space group: *R3*)^33^. Therefore, we have shown that the A_2_FeMoO_6_ not only have large magnetization and polarization, but also possess room-temperature magnetic transition temperature 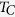. These encouraging properties make A_2_FeMoO_6_ a promising candidate for future multistate memory applications.

**Figure 5:**
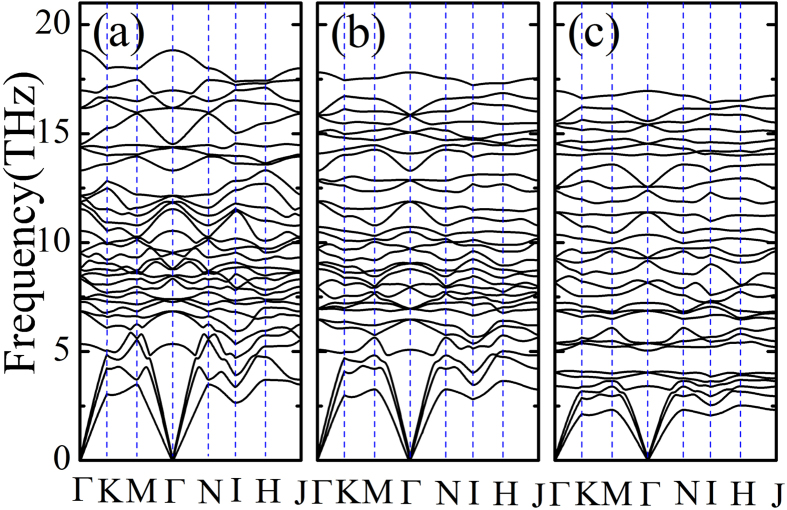
Phonon dispersion of Ni_3_TeO_6_-type A_2_FeMoO_6_ with 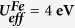, 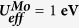, and 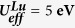. (**a**) ScFeO_3_. (**b**) Sc_2_FeMoO_6_. (**c**) Lu_2_FeMoO_6_. The wave vector takes a path along the high symmetrical points of the Brillouin Zone: Γ (0, 0, 0) → K (1/3, 1/3, 0) → M (1/2, 0, 0) → Γ (0, 0, 0) → N (0, 0, 1/2) → I (1/3, 1/3, 1/2) → H (1/2, 0, 1/2) → J (0, 0, 1/2).

It remains to be verified that the structure of ferrielectric and ferrimagnetic Ni_3_TeO_6_-type A_2_FeMoO_6_ (A = Sc, Lu) insulators are robust structures and can be prepared by the usual laboratory method. Therefore, the phonon dispersion spectra are calculated using the frozen-phonon method. The calculated phonon dispersions are plotted in Fig. 5 for both the reference compound ScFeO_3_ and Ni_3_TeO_6_-type A_2_FeMoO_6_ (A = Sc, Lu). The overall dispersion curves are quite similar for the three compounds except that the phonon frequency scales with the inverse square root of transition metal ion mass. It is clear that the soft-phonon modes are absent in the entire Brillouin Zone, which indicates that the Ni_3_TeO_6_-type A_2_FeMoO_6_ structure does correspond to stable structures.

To further check the stability of Ni_3_TeO_6_-type A_2_FeMoO_6_ (A = Sc, Lu) (*R3* structure) against other common structures, we have also considered 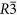, *P21/c*, and *C2* structures. After the full structural relaxation with respect to the atomic positions and lattice constants, the initial trial *C2* structure may converge either to *C2/m*, *C2*, *C2/c*, or *Imma* structure depending on the material composition. The calculated energies of different structures are summarized in Table 2. Only those of ferrimagnetic (antiferromagnetic) states are shown because they always have lower energy than those of ferromagnetic state. One finds that Ni_3_TeO_6_-type A_2_FeMoO_6_ (*R3* structure) consistently has lower energy than other structures. However, for large ionic radius of Y atom, the stable structure of Y_2_FeMoO_6_ takes *P21/c* space group rather than the *R3* space group. This suggests that Ni_3_TeO_6_-type A_2_FeMoO_6_ is stable with respect to *P21/c* structure only for small ionic radius of A atoms (see Supplementary Information Table S4). The paraelectric to ferrielectric transition temperature can also be estimated from the energy difference between the structurally connected polar (*R3*) and nonpolar 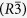 structures. As shown in Table 2, the energy difference is 1.378 eV/2f.u. for ScFeO_3_, 0.408 and 0.542 eV/f.u. for Sc_2_FeMoO_6_ and Lu_2_FeMoO_6_, respectively. Scaling the energy with that of ScFeO_3_ and considering its polar structure being stable above 1400 K^1^,^2^ yield a paraelectric-ferrielectric transition temperature 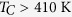 for Sc_2_FeMoO_6_ and 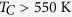 for Lu_2_FeMoO_6_. Both of them are well above room-temperature.

In the view that ScFeO_3_, Mn_2_FeMO_6_ (M = Nb, Ta, Mo, and W), and Ni_3_TeO_6_, all with smaller A-site ions, can be synthesized under the high temperature and high pressure environment^34^, we expect that the Ni_3_TeO_6_-type A_2_FeMoO_6_ can also be synthesized under similar conditions. If so, one expects that other room-temperature ferrielectric and ferrimagnetic insulators may also be realized in the corundum-derived transition metal oxides. Through incorporating different magnetic transition metal ions on the cation sites, one can easily tune the superexchange interaction and polar distortion, so that the polarization, magnetization, magnetoelectric coupling as well as critical temperature can be optimized for potential applications.

In summary, comprehensive first-principles calculations have been carried out for the structural, electronic, and magnetic properties of Ni_3_TeO_6_-type A_2_FeMoO_6_ (A = Sc, Lu). All of them show the ferrielectric and ferrimagnetic insulator properties with large magnetization (2μ_*B*_/f.u.) and polarization (>7 *μ*Ccm^−2^). The strong antiferromagnetic superexchange interaction between Fe and Mo yields a mean-field critical temperature above room-temperature. Strong intrinsic magnetoelectric coupling is also ensured because the magnetic ions are involved in both the magnetic moment formation and polarization. The Ni_3_TeO_6_-type Sc_2_FeMoO_6_ and Lu_2_FeMoO_6_ are also proved to be stable structures because they have lower energies than other possible structures. Thus, one expects that these materials and other related ones can be synthesized in experiments.

## Methods

The study has been carried out using the generalized gradient approximation + *U* (GGA + *U*) method^35^ with Perdew-Becke-Erzenhof exchange-correlation functional^36^ as implemented in the Vienna *ab Initio* simulation package (VASP)^37^,^38^. To account for the population imbalance on localized transition metal *d*- and rare earth *f*-orbitals, the effective on-site Coulomb interactions 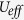 = 4.0, 1.0, and 5.0 eV are adopted for Fe-3*d*, Mo-4*d* and Lu-5*f* electrons, respectively^39^. The projector augmented wave (PAW) potentials^40^ explicitly include three valence electrons for Sc (*3d*^*1*^*4s*^*2*^), 11 for Y (*4s*^*2*^*4p*^*6*^*4d*^*1*^*5s*^*2*^), and 25 for Lu (*5s*^*2*^*5p*^*6*^*4f*^*14*^*5d*^*1*^*6s*^*2*^), 14 for Fe (*3p*^*6*^*3d*^*6*^*4s*^*2*^), 12 for Mo (*4p*^*6*^*4d*^*5*^*5s*^*1*^), and six for O (*2s*^*2*^*2p*^*4*^) atoms. The same result is also obtained for the PAW potential excluding *f* electrons for Lu. The wave function is expanded in a plane wave basis with an energy cutoff of 600 eV. The crystal unit cell includes two formula units for ScFeO_3_, and one formula unit for Sc_2_FeMoO_6_ and Lu_2_FeMoO_6_. A 7 × 7 × 7 Γ-centered *k*-points sampling is used for reciprocal space integrations. Each self-consistent electronic calculation is converged to 10^−6^ eV and the tolerance force is set to 0.005 eV/Å for ionic relaxation. The convergence checks with respect to the k-points sampling have been made for the total energy, densities of states as well as the phonon dispersion curves (see Supplementary Information Figures S1-S3).

To calculate the electric polarization of Ni_3_TeO_6_-type A_2_FeMoO_6_ (A = Sc, Lu) with space group *R3*, we choose the structure with space group 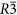 as a reference state^41^. The 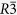 structure displayed in Figure S1 has space inversion symmetry. It is a non-polar insulator and has zero electric polarization (see Supplementary Information). Since the electric polarization is along 3-fold rotational axis, a 30-atom hexagonal unit cell is chosen, so that the in-plane polarization is zero. In calculating the electric polarization, a 7 × 7 × 4 Γ-centered *k*-points sampling is used for the self-consistent loop and 14 *k*-points sampling is adopted for parallel direction integration in Berry phase method. As shown in Figure S5, 14 k-points sampling is almost convergent for electric polarization calculation.

To calculate the phonon dispersion of Ni_3_TeO_6_-type structure of A_2_FeMoO_6_ (A = Sc, Lu) and ScFeO_3_, the structures are firstly atomically relaxed with a higher accuracy using the 8×8×8 Γ-centered *k*-points sampling and the tolerance force of 0.0001 eV/Å. The phonon dispersion is then calculated using the Phonopy code^42^ with a 2 × 2 × 2 supercell composed of ten-atom rhombohedral unit cell. The force constants are calculated by VASP using a 4 × 4 × 4 Γ-centered k-points sampling for the supercell.

## Additional Information

**How to cite this article**: Song, G. and Zhang, W. Comparative studies on the room-temperature ferrielectric and ferrimagnetic Ni_3_TeO_6_-type A_2_FeMoO_6_ compounds (A = Sc, Lu). *Sci. Rep.* **6**, 20133; doi: 10.1038/srep20133 (2016).

## Supplementary Material

Supplementary Information

## Figures and Tables

**Table 1:** The structural parameters and atomic positions for the ferrimagnetic state of Ni_3_TeO_6_-type A_2_FeMoO_6_ (space group: *R3*) and the antiferromagnetic state of ScFeO_3_ (space group: *R3c*) calculated with 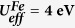 , 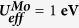 , and 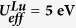 .

	Sc_2_FeMoO_6_(Theory)	Lu_2_FeMoO_6_(Theory)	ScFeO_3_(Theory)	ScFeO_3_ (Experiment)
*a/*Å	5.053	5.391	5.219	5.197
*c/*Å	13.511	14.330	14.027	13.936
*z*_*A1*_	0.1258	0.1220	0.1216	0.1228
*z*_*A2*_	0.2887	0.2853	0.2882	0.2895
*z*_*Fe*_	0.0000	0.0000	0.0000	0.0000
*z*_*Mo*_	0.1595	0.1604	0.1664	0.1667
*x*_*O1*_	0.3217	0.3073	0.3169	0.3172
*y*_*O1*_	0.3562	0.3534	0.3581	0.3555
*z*_*O1*_	0.0629	0.0638	0.0627	0.0622
*x*_*O2*_	0.3692	0.3805	0.3745	0.3716
*y*_*O2*_	0.0288	0.0262	0.0247	0.0221
*z*_*O2*_	0.2301	0.2309	0.2293	0.2288
Fe-O-Mo/°	135.17	137.82	135.71	135.13
d_A1_/Å	0.348	0.411	0.315	0.336
d_A2_/Å	0.294	0.357	0.315	0.336
d_Fe_/Å	0.276	0.277	0.261	0.314
d_Mo_/Å	0.183	0.187	0.261	0.314

The last column lists the available experimental data. *a* and *c* are the lattice constants in the hexagonal setting, whereas, x, y, and z are the reduced internal atomic positions of A(1) (0, 0, *z*), A(2) (0, 0, *z*), Fe (0, 0, *z*), Mo (0, 0, *z*), and O (*x*, *y*, *z*). Fe-O-Mo is the Fe-O-Mo angle. d_A(1)_, d_A(2)_, d_Fe_, and d_Mo_ refer to the atomic displacements with respect to neutral planes in Fig. 1(b,c).

**Table 2:** The relative stabilities of various phases of A_2_FeMoO_6_ calculated with 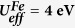 , 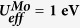 , and 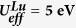 .

	*R3*	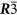	*P21/c*	*C2*
Sc_2_FeMoO_6_	0	0.408	0.519	0.676 (*C2/m*)
Lu_2_FeMoO_6_	0	0.524	0.004	0.606 (*C2*)
ScFeO_3_	0 (*R3c*)	1.378 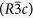	0.281 (*Pnma*)	1.024 (*Imma*)

The energy is given in unit of eV with *R3* phase taken as the reference structure.
