# Supplementary material for: Comparative studies on the room-temperature ferrielectric and ferrimagnetic Ni3TeO6-type A2FeMoO6 compounds (A = Sc, Lu)
Source: Sci Rep. 2016 Feb 1;6:20133. doi: 10.1038/srep20133 (PMC4735590; doi:10.1038/srep20133)
Supplement: Supplementary Information [file srep20133-s1.pdf]

Supplementary Information for "Comparative studies on the room-temperature ferrielectric and ferrimagnetic Ni_3TeO_6-type A_2FeMoO_6 compounds (A = Sc, Lu)"

Guang Song[1] & Weiyi Zhang[1, 2, *]

[1]*National Laboratory of Solid State Microstructures and Department of Physics, Nanjing University, Nanjing 210093, China*

[2]*Collaborative Innovation Center of Advanced Microstructures, Nanjing University, Nanjing 210093, China*

*Corresponding author: wyzhang@nju.edu.cn

The supplementary Information includes following materials:

(1) The convergence checks on k-points samplings for total energies, densities of states (DOS), and phonon dispersion curves;

(2) The $R\bar{3}$ reference structure and parameter setting for calculating the electric polarization using Berry phase method;

(3) The parallel calculations within GGA approach (U = 0) and the effect of effective on-site Coulomb repulsion U on electric polarization;

(4) The electric polarizations for both ferrimagnetic and ferromagnetic states calculated using Berry phase method;

(5) The relative stabilities of Ni_3TeO_6-type A_2FeMoO_6 (A = Sc, Lu, Y) in comparison with monoclinic *P21/c* or *C2* structures.

(1) The convergence checks on k-points samplings for total energies, densities of states (DOS), and phonon dispersion curves.

In total energy calculations we use a 7×7×7 Γ-centered *k*-points sampling, and the energy convergence check is displayed in Figure S1. In the DOS calculations, we use a 14×14×14 Γ-centered *k*-points sampling. In Figure S2, we plot the total DOSs with 7×7×7 and 14×14×14 *k*-points samplings, they virtually overlap each other. For the phonon dispersion curves, we have used, 3×3×3, 4×4×4, and 5×5×5 Γ-centered *k*-points samplings for the calculations, and the results are presented in Figure S3. No discernable differences are observed. Therefore, excellent numerical convergences have been achieved on the total energies, densities of states, and phonon dispersion curves.

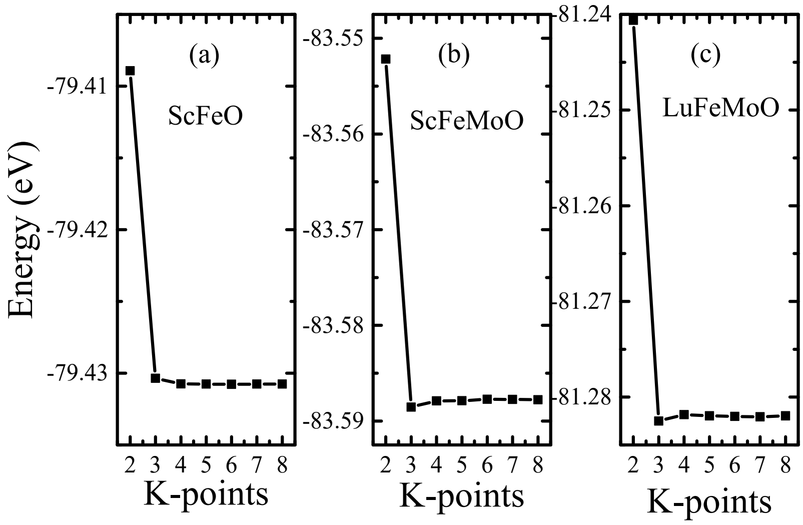

Figure S1. The convergence checks on k-points samplings for the total energies with $U_{eff}^{Fe} = 4$ eV, $U_{eff}^{Mo} = 1$ eV, and $U_{eff}^{Lu} = 5$ eV. (a) $ScFeO_3$; (b) Sc_2FeMoO_6; (c) Lu_2FeMoO_6.

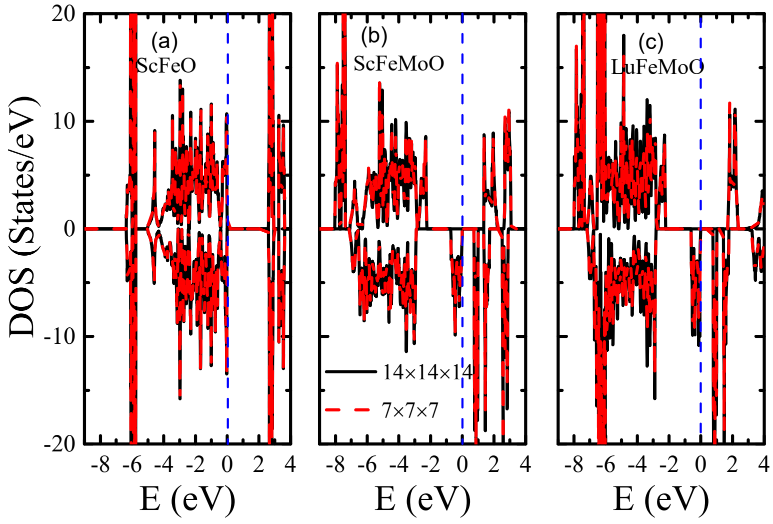

Figure S2. The convergence checks on k-points samplings for the total densities of states with $U_{eff}^{Fe} = 4$ eV, $U_{eff}^{Mo} = 1$ eV, and $U_{eff}^{Lu} = 5$ eV. The solid black and dashed red lines refer to those calculated with 14x14x14 and 7x7x7 k-points samplings. No discernable differences are observed. (a) $ScFeO_3$; (b) Sc_2FeMoO_6; (c) Lu_2FeMoO_6.

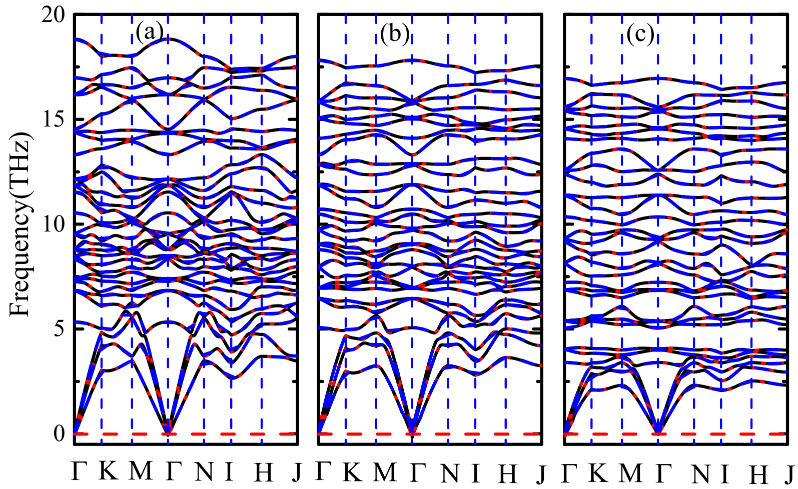

Figure S3. The convergence checks on k-points samplings for the phonon dispersion curves with $U_{eff}^{Fe} = 4$ eV, $U_{eff}^{Mo} = 1$ eV, and $U_{eff}^{Lu} = 5$ eV. The solid black, dashed red, and blue dotted lines refer to those calculated with 3×3×3, 4×4×4, 5×5×5 k-points samplings. Again, no discernable differences are observed. (a) $ScFeO_3$; (b) Sc_2FeMoO_6; (c) Lu_2FeMoO_6.

(2) The $R\bar{3}$ reference structure and parameter setting for calculating the electric polarization using Berry phase method:

To calculate the electric polarization of Ni_3TeO_6-type A_2FeMoO_6 (A = Sc, Lu) with space group *R3*, we choose the structure with space group $R\bar{3}$ as a reference state[1]. The $R\bar{3}$ structure displayed in Figure S4 has space inversion symmetry. It is a non-polar insulator and has zero electric polarization. Since the electric polarization is along 3-fold rotational axis, a 30-atoms hexagonal unit cell is chosen so that the in-plane polarization is zero. In calculating the electric polarization, a 7×7×4 Γ-centered k-points sampling is used for the self-consistent loop, and 14 k-points sampling is adopted for parallel direction integration in Berry phase method. As shown in Figure S5, 14 k-points sampling is almost convergent for electric polarization calculation.

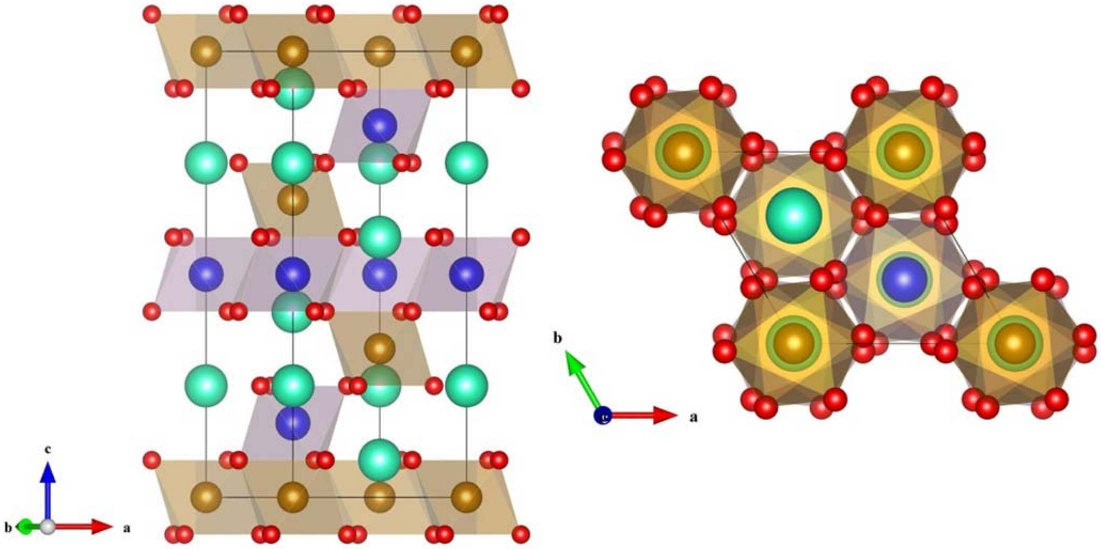

Figure S4. The $R\bar{3}$ structure of Ni_3TeO_6-type A_2FeMoO_6 (A = Sc, Lu) viewed along ($\bar{1}10$) direction (left) and (001) direction (right). The Fe and Mo-ions reside at the centers of FeO_6 and MoO_6 octahedra, respectively.

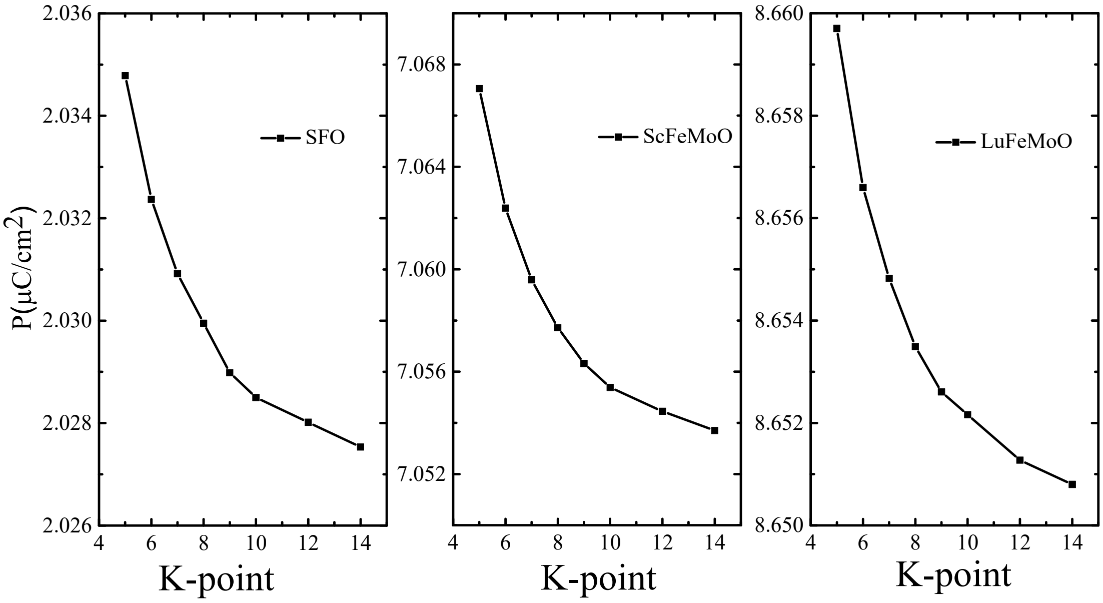

Figure S5. The convergence check on k-points samplings for the electric polarization calculated in Berry phase method with $U_{eff}^{Fe} = 4$ eV, $U_{eff}^{Mo} = 1$ eV, and $U_{eff}^{Lu} = 5$ eV.

(3) The parallel calculations within GGA approach (U = 0) and the effect of effective on-site Coulomb repulsion U on electric polarization:

For the pure GGA calculations, we find that ferrimagnetic (antiferromagnetic) state is consistently lower in energy than the ferromagnetic state. In fact, the ferromagnetic state is not even a metastable state except $ScFeO_3$. The initial trial ferromagnetic states always end up converging to the ferrimagnetic (antiferromagnetic) states for Ni_3TeO_6-type A_2FeMoO_6 (A = Sc, Lu) within GGA approach. The energy differences $\Delta E = E_{Ferro} - E_{Ferri}$ between the ferromagnetic state and ferrimagnetic (antiferromagnetic) state are listed in Table S1, and only the data for $ScFeO_3$ (0.715 eV) is available. As shown in Figure S6, the ferrimagnetic state of Ni_3TeO_6-type

A_2FeMoO_6 (A = Sc, Lu) and antiferromagnetic state of $ScFeO_3$ preserve the insulator nature though the band-gaps become smaller within GGA approach. The band-gap values are 0.104, 0.156, and 1.034 eV for Sc_2FeMoO_6, Lu_2FeMoO_6, and $ScFeO_3$, respectively.

Table S1. The energy difference $\Delta E = E_{Ferro} - E_{Ferri}$ and the magnetic moments of Fe and Mo ions in ferrimagnetic state of Ni_3TeO_6-type A_2FeMoO_6 with $U_{eff}^{Fe} = 0$ eV, $U_{eff}^{Mo} = 0$ eV, and $U_{eff}^{Lu} = 0$ eV.

	Sc_2FeMoO_6	Lu_2FeMoO_6	$ScFeO_3$
ΔE/eV	-	-	0.715
$\boldsymbol{\mu_{Fe}/\mu_B}$	3.635	3.590	3.722
$\boldsymbol{\mu_{Mo}/\mu_B}$	1.712	1.704	-
Band-gap/eV	0.104	0.156	1.034

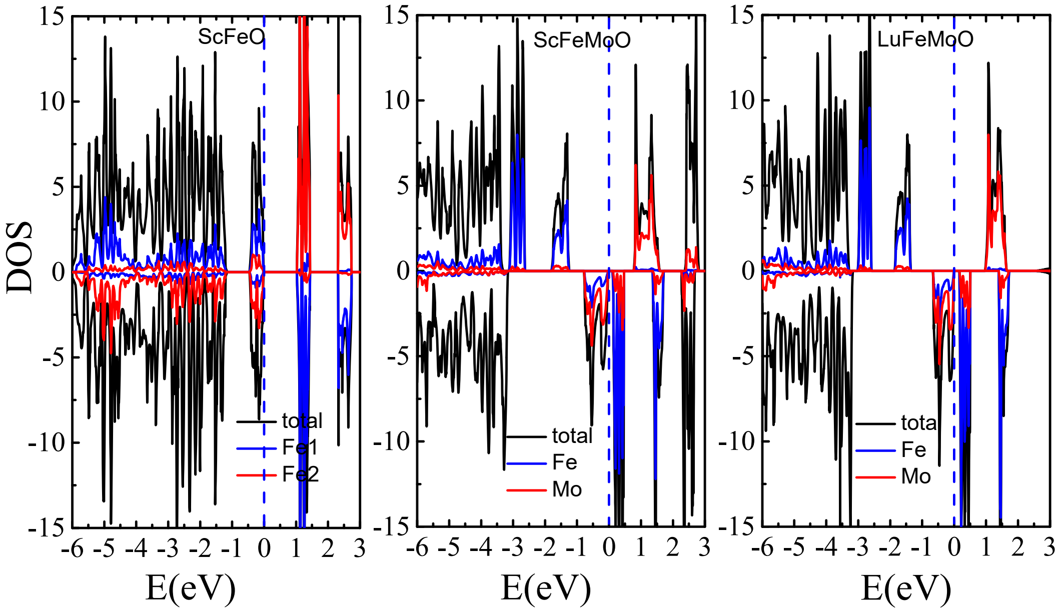

Figure S6. The total and partial densities of states of the ferrimagnetic state of Ni_3TeO_6-type

A_2FeMoO_6 (A = Sc, Lu) and antiferromagnetic state of $ScFeO_3$ calculated by GGA method with $U_{eff}^{Fe} = 0$ eV, $U_{eff}^{Mo} = 0$ eV, and $U_{eff}^{Lu} = 0$ eV.

In order to check the effect of effective on-site Coulomb repulsion U_{eff} on electric polarization, the electric polarizations are calculated with different U_{eff}, and are listed in Table S2. It is seen that the electric polarization increases with U_{eff}, but the dependence is not dramatic for A_2FeMoO_6. Also comparing with the experimentally measured electric polarization for $ScFeO_3$, U_{eff}^{Fe} should be larger than 3 eV.

Table S2. The effect of effective on-site Coulomb repulsion on electric polarizations. The electric polarization value is given in unit of μC/cm^2

	Sc_2FeMoO_6	Lu_2FeMoO_6	$ScFeO_3$
$U_{eff}^{Fe} = 3$ eV<br>$U_{eff}^{Mo} = 1$ eV<br>$U_{eff}^{Lu} = 5$ eV	5.098	7.444	0.464
$U_{eff}^{Fe} = 4$ eV<br>$U_{eff}^{Mo} = 1$ eV<br>$U_{eff}^{Lu} = 5$ eV	7.054	8.651	2.027
$U_{eff}^{Fe} = 4$ eV<br>$U_{eff}^{Mo} = 2$ eV<br>$U_{eff}^{Lu} = 5$ eV	8.103	9.673	2.027

(4) The electric polarizations for both ferrimagnetic and ferromagnetic states calculated using Berry phase method:

The electric polarizations of both ferrimagnetic and ferromagnetic states for Ni_3TeO_6-type A_2FeMoO_6 (A = Sc, Lu) and $ScFeO_3$ are calculated using Berry phase method within GGA + U approach. The structure with $R\bar{3}$ space group is chosen as a non-polar reference structure and 14 k-points sampling is used for the parallel direction integration in Berry phase method. As seen in Table S3, the electric

polarization values are on the same orders of magnitude for ferrimagnetic and ferromagnetic states. The values are slightly larger for ferromagnetic state in Sc_2FeMoO_6 and Lu_2FeMoO_6, and for antiferromagnetic state in $ScFeO_3$. The magnetic transition from ferrimagnetic to ferromagnetic states can probably be induced via external electric field.

Table S3. The electric polarizations of Ni_3TeO_6-type A_2FeMoO_6 for the ferrimagnetic and ferromagnetic states. The electric polarization value is given in unit of μC/cm^2 with $U_{eff}^{Fe} = 4$ eV, $U_{eff}^{Mo} = 1$ eV, and $U_{eff}^{Lu} = 5$ eV.

	Sc_2FeMoO_6	Lu_2FeMoO_6	$ScFeO_3$
Ferrimagnetic state	7.054	8.651	2.027
Ferromagnetic state	8.118	11.588	1.627

(5) The relative stability of Ni_3TeO_6-type A_2FeMoO_6 (A = Sc, Lu, Y) in comparison with monoclinic *P21/c* or *C2* structures:

We have considered $R\bar{3}$, *P21/c*, and *C2* structures in addition to Ni_3TeO_6-type A_2FeMoO_6 (*R3* structure). After the full structural relaxation with respect to the atomic positions and lattice constants, the initial trial *C2* structure may converge either to *C2/m*, *C2*, *C2/c*, or *Imma* structure depending on the material composition. The calculated energies of different structures are summarized in Table S3. Only those of ferrimagnetic (antiferromagnetic) states are shown because they always have lower energy than those of ferromagnetic state. One finds that Ni_3TeO_6-type Sc_2FeMoO_6

and Lu_2FeMoO_6 (*R3* structure) consistently have lower energy than other structures while Y_2FeMoO_6 has lower energy in *P21/c* structure rather than in *R3* structure. This suggests that Ni_3TeO_6-type A_2FeMoO_6 is stable with respect to *P21/c* structure only for small ionic radius of A atoms. This also suggests that metastable Ni_3TeO_6-type Y_2FeMoO_6 with relatively larger cell volume (Ω = 125.93 Å^3) may be driven into *P21/c* structure with relatively smaller cell volume (Ω = 121.89 Å^3) under high pressure. Such structural transition has indeed been observed in Mn_2FeSbO_6 where the metastable *R3* structure is prepared under 3 GPa pressure and 1000 °C, and can make transition to *P21/c* structure if annealed under 6 GPa pressure and 1000 °C[2].

Table S4. The relative stabilities of various phases of A_2FeMoO_6 using $U_{eff}^{Fe} = 4$ eV, $U_{eff}^{Mo} = 1$ eV, and $U_{eff}^{Lu} = 5$ eV. The energy is given in unit of eV with *R3* phase taken as the reference structure.

	R3	$R\bar{3}$	*P21/c*	*C2*
Sc_2FeMoO_6	0	0.408	0.519	0.676 (*C2/m*)
Lu_2FeMoO_6	0	0.524	0.004	0.606 (*C2*)
Y_2FeMoO_6	0	0.288	-0.531	0.573 (*C2/c*)
$ScFeO_3$	0 (*R3c*)	1.378 ($R\bar{3}c$)	0.281 (*Pnma*)	1.024 (*Imma*)

Table S5. The structural parameters of A_2FeMoO_6 (A=Sc, Lu, Y) in $R\bar{3}$ phase and $ScFeO_3$ in $R\bar{3}c$ phase using $U_{eff}^{Fe} = 4$ eV, $U_{eff}^{Mo} = 1$ eV, and $U_{eff}^{Lu} = 5$ eV. The fractional coordinates of Fe and Mo are (0.0, 0.0, 0.0) and (0.5, 0.5, 0.5) in A_2FeMoO_6 and (0.0, 0.0, 0.0) for Fe in $ScFeO_3$.

	Sc_2FeMoO_6	Lu_2FeMoO_6	Y_2FeMoO_6	$ScFeO_3$

a	5.3828	5.5208	5.5389	5.3443
c	14.4625	14.0967	14.2185	13.1546
O_x	0.0240	0.0366	0.0550	0.0411
O_y	0.7059	0.7171	0.7279	0.7078
O_z	0.0946	0.0941	0.0903	0.0833

Table S6. The structural parameters of A_2FeMoO_6 (A=Sc, Lu, Y) in *P21/c* phase and $ScFeO_3$ in *Pnma* phase using $U_{eff}^{Fe} = 4$ eV, $U_{eff}^{Mo} = 1$ eV, and $U_{eff}^{Lu} = 5$ eV. The fractional coordinates of Fe and Mo are (0.50, 0.00, 0.25) and (0.00, 0.50, 0.25) in A_2FeMoO_6 and (0.50, 0.00, 0.25) for Fe in $ScFeO_3$.

	Sc_2FeMoO_6	Lu_2FeMoO_6	Y_2FeMoO_6	$ScFeO_3$
a	5.1338	5.2686	5.3724	5.0567
b	5.4582	5.6285	5.8378	5.3896
c	7.7674	7.8528	7.7740	7.6782
Sc_x	0.5260	0.5269	0.5254	0.5283
Sc_y	0.4334	0.4300	0.4259	0.4318
Sc_z	0.0087	0.0095	0.0017	0.000
$O1_x$	0.8136	0.8017	0.8152	0.8118
$O1_y$	0.1874	0.1934	0.1938	0.1921
$O1_z$	0.1640	0.1632	0.1819	0.1732
$O2_x$	0.3455	0.3507	0.3688	0.3514
$O2_y$	0.0773	0.0721	0.0554	0.0678

O2$_z$	-0.0020	-0.0127	-0.0075	0.000
O3$_x$	0.6755	0.6898	0.6959	0.6882
O3$_y$	-0.3102	-0.3208	-0.3185	-0.3079
O3$_z$	0.1800	0.1897	0.1916	0.1732

Table S7. The structural parameters of Sc_2FeMoO_6 in *C2/m* phase using $U_{eff}^{Fe} = 4$ eV and $U_{eff}^{Mo} = 1$ eV. (a = 9.9217 Å, b = 5.5940, c = 5.0321, β = 60.2781°).

	x	*y*	*z*
Sc	0.7450	0.0000	0.8301
Fe	0.0000	0.0000	0.0000
Mo	0.0000	0.5000	0.5000
O1	0.2952	0.0000	0.5164
O2	0.4043	0.2511	0.8740

Table S8. The structural parameters of Lu_2FeMoO_6 in *C2* phase with using $U_{eff}^{Fe} = 4$ eV, $U_{eff}^{Mo} = 1$ eV, and $U_{eff}^{Lu} = 5$ eV. (a = 10.2916 Å, b = 5.3622 Å, c = 5.1594 Å, β = 119.1379°).

	x	*y*	*z*
Lu	0.2466	0.0336	0.7052
Fe	0.0000	0.0062	0.0000
Mo	0.0000	0.4843	0.5000
O1	0.1995	0.4260	0.8611

O2	0.4085	0.2584	0.6519
O3	0.0993	0.2625	0.3189

Table S9. The structural parameters of Y_2FeMoO_6 in *C2/c* phase with $U_{eff}^{Fe} = 4$ eV and $U_{eff}^{Mo} = 1$ eV (*a* = 9.8991 Å, *b* = 5.6608 Å, *c* = 9.8172 Å, *β* = 113.7449°).

	x	*y*	*z*
Y	0.6451	0.1930	0.6406
Fe	0.0000	0.2240	0.7500
Mo	0.7500	0.2500	0.0000
O1	0.9278	0.2827	0.9456
O2	0.8465	0.0379	0.1848
O3	0.6757	0.0322	0.3611

Table S10. The structural parameters of $ScFeO_3$ in *Imma* phase with $U_{eff}^{Fe} = 4$ eV. (*a* = 7.9043 Å, *b* = 5.4271 Å, *c* = 5.0992 Å).

	x	*y*	*z*
Sc	0.0000	0.2500	0.1996
Fe	0.2500	0.2500	0.7500
O1	0.5000	0.7500	0.8098
O2	0.1655	1.0000	0.0000

1. Resta, R. Macroscopic polarization in crystalline dielectrics: the geometric phase approach. *Rev.*

Mod. Phys. **66,** 899 (1994).

2. Mathieu, R. *et al*. Mn_2FeSbO_6: A ferrimagnetic ilmenite and an antiferromagnetic perovskite. *Phys. Rev. B* **87,** 014408 (2013).
